# Impact of Disability Status on Mortality in Patients with Gastric Cancer: A Nationwide Study Focusing on Regional Disparities

**DOI:** 10.3390/healthcare11050641

**Published:** 2023-02-21

**Authors:** Woo-Ri Lee, Kyu-Tae Han, Mingee Choi, Seojin Park, Woorim Kim

**Affiliations:** 1 Division of Cancer Control & Policy, National Cancer Control Institute, National Cancer Center, Goyang-si 10408, Republic of Korea; 2 Department of Healthcare Management, Graduate School of Public Health, Yonsei University, Seoul 03722, Republic of Korea

**Keywords:** disability, mortality, region, gastric cancer, disparity

## Abstract

Background: Disparities in mortality according to disability status require investment, as individuals with disabilities form the largest subset of the vulnerable population. This study aimed to investigate the association between mortality and disability status in patients with gastric cancer as well as how regional disparities modify this relationship. Methods: Data were obtained from the National Health Insurance claims database in South Korea for the period of 2006–2019. The outcome measures were all-cause 1-year, 5-year, and overall mortality. The main variable of interest was disability status, categorized into “no disability”, “mild disability”, and “severe disability”. A survival analysis based on the Cox proportional hazards model was conducted to analyze the association between mortality and disability status. Subgroup analysis was conducted according to region. Results: Of the 200,566 study participants, 19,297 (9.6%) had mild disabilities, and 3243 (1.6%) had severe disabilities. Patients with mild disabilities had higher 5- and overall mortality risks, and those with severe disabilities had higher 1-year, 5-year, and overall mortality risks than those without disabilities. These tendencies were generally maintained regardless of the region, but the magnitude of the differences in the mortality rates according to disability status was higher in the group residing in non-capital regions than in the group living in the capital city. Conclusion: Disability status was associated with all-cause mortality in patients with gastric cancer. The degree of the differences in mortality rates among those with “no disability”, “mild disability”, and “severe disability” was augmented in the group residing in non-capital regions.

## 1. Introduction

Globally, gastric cancer is the fifth most prevalent cancer and the fourth leading cause of cancer-related mortality in 2020 [1]. Although the incidence and mortality of gastric cancer have been decreasing in many parts of the world, their incidence varies noticeably across the globe and is the highest in East Asian countries, including South Korea [2,3]. Gastric cancer is the most frequently diagnosed cancer and is projected to be the fourth leading cause of cancer-related death in men [4,5]. The five-year survival rate of patients with gastric cancer in South Korea is relatively high but this rate declines as the cancer stage advances [6,7]. Considering the significance of gastric cancer in East Asian countries, there is a need to explore factors associated with gastric cancer mortality.

Among the many factors associated with gastric cancer mortality, disability status requires attention, as individuals with disabilities form the largest subset of the vulnerable population [8]. Around 15.6% of the world’s population is reported to have disabilities, and in the case of South Korea, which implements a narrow definition of disability, the prevalence rate of disability was approximately 5.4% in 2017 [8]. People with disabilities often have poor access to healthcare services because they tend to face various physical, psychosocial, or other practical barriers [8,9]. Individuals with disabilities are also less likely to have their medical needs met on a regular basis, despite needing more recurrent services than the general population [10,11]. Previous studies have shown that disability is a predictive factor for all-cause mortality [12]. Unsurprisingly, cancer patients, including those with gastric cancer, who also have disabilities tend to have poorer survival rates as they are less likely to receive screening, be diagnosed at an earlier stage, or receive appropriate treatment than those who do not have disabilities [8,13,14]. 

The region of residence is an important factor to consider in the association between disability status and mortality in cancer patients, as one of the most commonly reported reasons for unmet healthcare needs is difficulty in obtaining reliable transportation [11]. In fact, people with disabilities often report lower satisfaction with their medical care than those without disabilities, with physically inaccessible healthcare settings and a lack of dependable transportation being cited as major influencing factors [15]. Considering that such barriers are related to the environment, individuals with disabilities diagnosed with gastric cancer residing in remote regions may have poorer outcomes than those residing in the capital area because tertiary hospitals and related medical resources tend to be highly concentrated in the capital area in South Korea [16]. 

This study aimed to investigate the impact of disability status on 1-year and 5-year all-cause mortality and overall mortality in adult patients with gastric cancer. The disability status was categorized into “no disability”, “mild disability”, and “severe disability”. A subgroup analysis was conducted based on region to explore how this factor modifies the relationship between disability status and mortality in patients with gastric cancer. 

## 2. Methods

### 2.1. Data and Study Population

This study used data from the National Health Insurance (NHI) claims database. This retrospective cohort data included information on the medical claims of individuals diagnosed with gastric cancer (International Classification of Diseases, 10th edition (ICD-10) code C16). Of the 210,993 individuals aged ≥19 years who were first diagnosed with gastric cancer between 2007 and 2015, those who did not receive any treatment (*n* = 10,186) and who died within a month of cancer diagnosis (*n* = 241) were excluded, resulting in a final study population of 200,566 individuals. 

### 2.2. Outcome Measures

The outcome measures of this study were all-cause, 1-year, 5-year, and overall mortality. The date of gastric cancer diagnosis (C16) and the date of receipt of cancer-specific health insurance (claims code V193) were identified as index dates. Survival time was defined as the period between the index date and the end of the observation period (death or censoring).

### 2.3. Independent Variables

The primary independent variables of this study were the categories of “no disability”, “mild disability”, and “severe disability”. Disability status was identified according to the Certificate of Persons with Disability—this certificate is issued by the certification service managed by the government to register people with disabilities after examination [17]. The severity level of disability varies, in which severe disability (levels 1–3) indicate dependence on assistance or assistive devices and mild disability (levels 4–6) the need of partial assistance [18].

The following covariates were included in the analysis: sex, age, income level, type of healthcare insurance, region, comorbidity status measured using the Charlson Comorbidity Index (CCI), type of cancer treatment, and type of hospital visited for cancer treatment. The CCI is a well-established and validated index for measuring comorbidity scores [19].

### 2.4. Statistical Analysis

A chi-square test was used to examine the general characteristics of the study population. The survival times between groups depending on disability status were compared using Kaplan–Meier survival curves and the log-rank test. The Cox proportional hazards model was used to conduct survival analysis, with adjustment for all the covariates. Subgroup analysis was conducted according to region, categorized into “capital area” and “non-capital regions”. All *p*-values were two-sided and considered significant at *p* < 0.05. Statistical analyses were conducted using the SAS statistical software (version 9.4; Cary, NC, USA).

## 3. Results

The general characteristics of the study population are summarized in Table 1. Of the 200,566 patients diagnosed with gastric cancer, 19,297 (9.6%) had a mild disability, and 3243 (1.6%) had a severe disability. In a step-wise manner, the 1-year (none: 4.4%; mild: 5.1%; severe: 8.5%); 5-year (none: 23.2%; mild: 26.7%; severe: 37.7%); and overall (none: 34.1%; mild: 43.1%; severe: 57.0%) all-cause mortality rates were higher in the participants with mild or severe disabilities than in those without disabilities.

**Figure 1:**
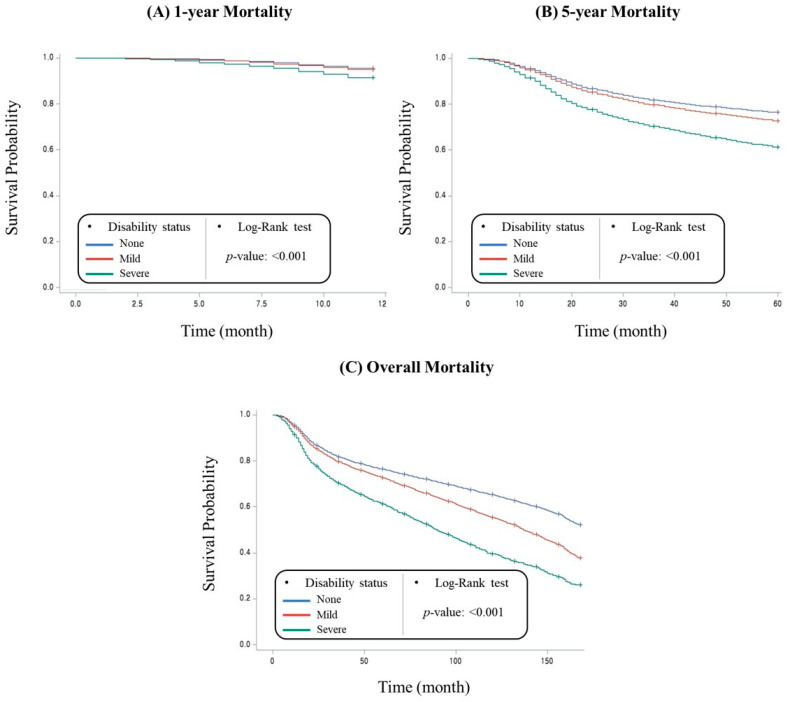
Kaplan-Meier survival curves for all-cause 1-year, 5-year, and overall mortality according to disability status.

The Kaplan–Meier survival curves plotted according to disability status are shown in Figure 1. Differences were found in the 1-year (*p* < 0.001), 5-year (*p* < 0.001), and overall mortality (*p* < 0.001) among patients with no disabilities, mild disabilities, and severe disabilities. The results of the survival analysis conducted with gastric cancer using multivariate adjustment are shown in Table 2. Those with mild disabilities showed higher risks of 5-year (hazard ratio [HR], 1.07; 95% confidence interval [CI], 1.04–1.11) and overall (HR, 1.12; 95% CI, 1.09–1.14) mortality. Patients with severe disabilities had increased risks of 1-year (HR, 1.61; 95% CI, 1.42–1.81), 5-year (HR, 1.62; 95% CI, 1.53–1.71), and overall (HR, 1.64; 95% CI, 1.57–1.72) mortality. 

The results of the subgroup analysis based on region are shown in Table 3. The tendencies shown in the main findings were generally maintained regardless of region. The magnitude of the differences in mortality according to disability status was higher in the individuals residing in the non-capital regions than in those living in the capital city. Such tendencies were found for 1-year (non-capital regions—severe: HR, 1.69; 95% CI, 1.48–1.92), 5-year (capital city—severe: HR, 1.46; 95% CI, 1.26–1.69 vs. non-capital regions—mild: HR 1.08; 95% CI, 1.05–1.12; severe: HR, 1.65; 95% CI, 1.55–1.76), and overall mortality (capital city—mild: HR, 1.12; 95% CI, 1.06–1.19; severe: HR, 1.45; 95% CI, 1.28–1.63 vs. non-capital regions—mild: HR, 1.11; 95% CI, 1.09–1.14; severe: HR, 1.68; 95% CI, 1.60–1.77). 

## 4. Discussion

The findings of this study revealed that the risk of all-cause mortality was comparatively higher in patients with gastric cancer and disabilities, particularly severe disabilities, than in those without disabilities. Specifically, a higher risk of short-term (1-year) mortality was observed in individuals with severe disabilities, whereas higher risks of long-term (5-year and overall) mortality were observed in patients with mild and severe disabilities than in those without disabilities. Such tendencies were generally maintained regardless of the patients’ region of residence, although the degree of differences in the risk of mortality according to disability status was augmented in participants living in non-capital areas than in those living in the capital city.

These results are in accordance with those of previous studies that investigated the impact of disability on cancer-related mortality. A study of Medicare beneficiaries in the United States revealed that patients with cancer and disabilities had higher all-cause mortality than those with cancer but no disabilities. Cancer-related mortality was also found to be more prevalent among adults with intellectual disabilities than among those without such disabilities in the Dutch population [20]. Regarding South Korea, a study involving patients with lung cancer concluded that patients with disabilities, especially those with severe disabilities, had a slightly higher mortality rate than their counterparts without disabilities [13]. Likewise, patients with cervical cancer and disabilities, particularly severe disabilities, were more likely to be diagnosed at later stages and have higher mortality rates than those without disabilities [21]. Another study also reported that patients with cancer and disabilities had higher all-cause long-term mortality than those without disabilities [22]. Similar tendencies were found in studies on gastric cancer, in which patients with disabilities showed an increased risk of overall mortality, with such tendencies being more prominent in those with severe disabilities [8].

Both mild and severe disabilities were associated with higher risks of all-cause 5-year and overall mortality, whereas only severe disability status was correlated with increased 1-year mortality in patients with gastric cancer. Such tendencies may have been partially influenced by the characteristics of the South Korean healthcare system, wherein all the people are covered by either the NHI or Medical Aid. The copayment level for covered services related to cancer is relatively low at only 5%, with a maximum cap for low-income patients [13]. In the case of NHI beneficiaries located within the lower 50% income level and Medical Aid beneficiaries, the National Cancer Screening Program is also available free of charge in addition to additional financial aid programs for cancer [23]. As patients with disabilities are highly likely to be recipients of such cancer control policies, disability-related gaps in the utilization of preventive cancer services and initial treatment may have been reduced for those with comparatively mild disabilities, which in turn may have helped reduce the disparities in short-term mortality [22]. 

The results of this study also show that the degree of differences in the risk of mortality by disability status in patients with gastric cancer differs according to the region of residence. This tendency may have been influenced by the high concentration of resources in the capital area of South Korea, where approximately half the population resides in the capital city of Seoul and nearby regions. The centralization of resources is also found in the medical sector, with patients with cancer favoring large tertiary hospitals in Seoul, given the accumulation of medical personnel and resources in the capital city [24]. Such regional disparities in access to different types of healthcare services are reported to be particularly magnified for individuals with disabilities, influenced by a variety of factors, such as the lack of limited public transportation and challenging environmental factors, given the lack of visual or auditory aids for people with disabilities in rural settings [15,25]. Access to medical services is a concern for individuals residing in rural areas worldwide, and this may be particularly problematic for patients with disabilities as hospitals in rural areas also often lack the resources needed to effectively care for individuals with severe or complex disabilities [26,27]. The findings may have also been influenced by the tendency of patients requiring active treatment, including surgery, to concentrate in large medical institutions located in large cities [28]. Hence, the high proportion of patients receiving treatment in the capital area may have affected the study results. Together, these findings suggest the need to consider regional disparities when addressing the health outcomes of patients with cancer according to their disability status. A need exists to enhance access to cancer care and assure better tailoring of screening, diagnosis, and treatment for patients with disabilities in the future.

This study had some limitations. First, the cancer stage at diagnosis could not be incorporated into the analyses owing to data limitations. To increase the homogeneity of the study population and partially overcome this limitation, individuals who died within one month of diagnosis or did not receive treatment for cancer within six months of diagnosis were excluded. Still, the differences found in mortality according to disability status may be affected by cancer severity and require in-depth investigation in the future. Second, this study only investigated all-cause mortality owing to data limitations. Further studies that consider cancer-specific mortality would be beneficial in providing further insights into this subject. Third, information on several potential covariates, such as education level and health literacy, was not available because this study used claims data. The analyses were adjusted for various sociodemographic and health-related factors recorded in the data used. Despite the limitations described above, this study is unique as it is one of the few studies to investigate the association between disability status and all-cause mortality in East Asian patients with gastric cancer using nationwide data as well as how regional factors may potentially interact with the stated relationship. 

## 5. Conclusions

Disability status was associated with all-cause mortality in patients with gastric cancer. Patients with gastric cancer and a mild disability had higher 5-year and overall mortality risks, whereas individuals with a severe disability had higher 1-year, 5-year, and overall mortality risks than the patients without disabilities. These tendencies were generally retained regardless of region, but the degree of the differences in all-cause mortality among those with “no disability”, “mild disability”, and “severe disability” was magnified in the group residing in non-capital regions. These findings suggest the importance of addressing disability-related and regional disparities in addressing the health outcomes of patients with gastric cancer. Future research on the potential methods to reduce such disparities through better tailoring of screening, diagnosis, and treatment and enhancing access to care is needed.

## Figures and Tables

**Table 1:** General characteristics of the study population.

Variables	Total	1-Year Mortality			5-Year Mortality			Overall Mortality		
		N	(%)	*p*-Value	N	(%)	*p*-Value	N	(%)	*p*-Value
**Disability status**										
No	178,026	7786	(4.4)	<0.001	41,333	(23.2)	<0.001	60,707	(34.1)	<0.001
Mild	19,297	989	(5.1)		5161	(26.7)		8316	(43.1)	
Severe	3243	275	(8.5)		1224	(37.7)		1850	(57.0)	
**Sex**										
Male	136,611	6481	(4.7)	<0.001	34,046	(24.9)	<0.001	51,276	(37.5)	<0.001
Female	63,955	2569	(4.0)		13,672	(21.4)		19,597	(30.6)	
**Age**										
19–49	34,217	1309	(3.8)	<0.001	7415	(21.7)	<0.001	8953	(26.2)	<0.001
50–59	50,324	1710	(3.4)		9775	(19.4)		12,896	(25.6)	
60–69	58,489	2189	(3.7)		12,342	(21.1)		18,965	(32.4)	
70–79	48,402	2826	(5.8)		14,146	(29.2)		23,859	(49.3)	
≥80	9134	1016	(11.1)		4040	(44.2)		6200	(67.9)	
**Income**										
Low	41,041	2210	(5.4)	<0.001	11,050	(26.9)	<0.001	16,379	(39.9)	<0.001
Low–middle	41,223	1969	(4.8)		10,313	(25.0)		14,732	(35.7)	
Middle–high	59,765	2532	(4.2)		13,772	(23.0)		20,295	(34.0)	
High	58,537	2339	(4.0)		12,583	(21.5)		19,467	(33.3)	
**Type of healthcare insurance**										
Medical Aid	8470	651	(7.7)	<0.001	2890	(34.1)	<0.001	4551	(53.7)	<0.001
NHI * Self employed	66,347	3269	(4.9)		16,888	(25.5)		24,242	(36.5)	
NHI * Employee	125,749	5130	(4.1)		27,940	(22.2)		42,080	(33.5)	
**Region**										
Capital city	126,267	5577	(4.4)	0.007	29,645	(23.5)	<0.001	43,247	(34.3)	<0.001
Other areas	74,299	3473	(4.7)		18,073	(24.3)		27,626	(37.2)	
**Charlson Comorbidity Index**										
0	54,753	407	(0.7)	<0.001	5633	(10.3)	<0.001	10,702	(19.5)	<0.001
1	24,806	339	(1.4)		3311	(13.3)		6843	(27.6)	
2	33,826	500	(1.5)		4766	(14.1)		8696	(25.7)	
≥3	87,181	7804	(9.0)		34,008	(39.0)		44,632	(51.2)	
**Type of treatment**										
Surgery only	147,392	3010	(2.0)	<0.001	15,750	(10.7)	<0.001	33,524	(22.7)	<0.001
Surgery and Chemo or radiotherapy	39,705	2403	(6.1)		20,239	(51.0)		25,441	(64.1)	
Chemo or radiotherapy only	13,469	3637	(27.0)		11,729	(87.1)		11,908	(88.4)	
**Type of hospital**										
Tertiary hospital	140,015	5585	(4.0)	<0.001	31,038	(22.2)	<0.001	46,407	(33.1)	<0.001
General hospital	60,551	3465	(5.7)		16,680	(27.5)		24,466	(40.4)	
**Total**	200,566	9050	(4.5)		47,718	(23.8)		70,873	(35.3)	

* NHI: National Health Insurance.

**Table 2:** The association between disability status and all-cause mortality in patients with gastric cancer.

Variables	1-Year Mortality			5-Year Mortality			Overall Mortality		
	HR *	95% CI *		HR *	95% CI *		HR *	95% CI *	
Disability status									
None	1.00			1.00			1.00		
Mild	1.04	(0.97)	(1.11)	1.07	(1.04)	(1.11)	1.12	(1.09)	(1.14)
Severe	1.61	(1.42)	(1.81)	1.62	(1.53)	(1.71)	1.64	(1.57)	(1.72)
Sex									
Male	1.00			1.00			1.00		
Female	0.88	(0.84)	(0.92)	0.89	(0.87)	(0.90)	0.79	(0.78)	(0.81)
Age									
19–49	1.00			1.00			1.00		
50–59	0.93	(0.87)	(1.00)	0.92	(0.89)	(0.95)	0.99	(0.96)	(1.01)
60–69	1.03	(0.96)	(1.11)	1.03	(1.00)	(1.06)	1.30	(1.27)	(1.33)
70–79	1.69	(1.58)	(1.81)	1.74	(1.69)	(1.80)	2.57	(2.51)	(2.63)
≥80	3.82	(3.50)	(4.16)	3.91	(3.76)	(4.07)	5.62	(5.44)	(5.82)
Income									
Low	1.00			1.00			1.00		
Low–middle	0.98	(0.91)	(1.05)	0.97	(0.95)	(1.00)	0.99	(0.96)	(1.01)
Middle–high	0.91	(0.85)	(0.96)	0.92	(0.90)	(0.95)	0.92	(0.89)	(0.94)
High	0.85	(0.80)	(0.91)	0.86	(0.84)	(0.89)	0.85	(0.83)	(0.87)
Type of healthcare insurance									
Medical Aid	1.00			1.00			1.00		
NHI Self employed	0.91	(0.82)	(1.00)	0.97	(0.93)	(1.02)	0.91	(0.88)	(0.95)
NHI Employee	0.80	(0.73)	(0.88)	0.88	(0.85)	(0.92)	0.84	(0.81)	(0.87)
Region									
Capital city	1.00			1.00			1.00		
Other areas	1.00	(0.96)	(1.05)	1.01	(0.99)	(1.03)	1.02	(1.00)	(1.03)
Charlson Comorbidity Index									
0	1.00			1.00			1.00		
1	1.67	(1.44)	(1.93)	1.25	(1.19)	(1.30)	1.29	(1.25)	(1.33)
2	1.75	(1.53)	(1.99)	1.23	(1.18)	(1.28)	1.24	(1.20)	(1.27)
≥3	6.08	(5.49)	(6.73)	2.51	(2.43)	(2.58)	2.12	(2.07)	(2.17)
Type of treatment									
Surgery only	1.00			1.00			1.00		
Surgery and Chemo or radiotherapy	2.59	(2.45)	(2.73)	6.34	(6.20)	(6.48)	4.58	(4.51)	(4.66)
Chemo or radiotherapy only	9.44	(8.97)	(9.94)	22.03	(21.45)	(22.63)	17.37	(16.96)	(17.78)
Type of hospital									
Tertiary hospital	1.00			1.00			1.00		
General hospital	1.21	(1.16)	(1.26)	1.12	(1.10)	(1.14)	1.08	(1.07)	(1.10)

* HR: Hazard Ratio; CI: Confidence Interval; NHI: National Health Insurance.

**Table 3:** Results of the subgroup analysis, stratified by region.

Subgroup		1-Year Mortality			5-Year Mortality			Overall Mortality		
		HR *	95% CI *		HR *	95% CI *		HR *	95% CI *	
Region	Disability status									
Capital city	None	1.00			1.00			1.00		
	Mild	0.96	(0.81)	(1.14)	1.04	(0.96)	(1.12)	1.12	(1.06)	(1.19)
	Severe	1.23	(0.89)	(1.70)	1.46	(1.26)	(1.69)	1.45	(1.28)	(1.63)
Other areas	None	1.00			1.00			1.00		
	Mild	1.05	(0.98)	(1.13)	1.08	(1.05)	(1.12)	1.11	(1.09)	(1.14)
	Severe	1.69	(1.48)	(1.92)	1.65	(1.55)	(1.76)	1.68	(1.60)	(1.77)

* HR: Hazard Ratio; CI: Confidence Interval.

## Data Availability

Data can be obtained after application and approval by the National Health Insurance Service.
